# Proficiency-based simulation training in healthcare – a scoping review protocol

**DOI:** 10.1007/s11845-026-04325-y

**Published:** 2026-04-02

**Authors:** Lorcan Jarlath O’Carroll, Don Walsh, Siobhan Scarlett, Mary F. Higgins, Robert Ffrench-O’Carroll

**Affiliations:** 1 National Maternity Hospital, Dublin, Ireland; 2 UCD School of Medicine and Medical Sciences, University College Dublin, Dublin, Ireland; 3 The Irish Longitudinal Study on Ageing (TILDA), Medical Gerontology, School of Medicine, Trinity College Dublin, Dublin, Ireland; 4 UCD Perinatal Research Centre, Obstetrics and Gynaecology, University College Dublin, National Maternity Hospital, Dublin, Ireland

**Keywords:** Education, Simulation, Proficiency-based training, Methodology

## Abstract

**Introduction:**

Proficiency-based training (PBT) is a novel approach for accelerating skills acquisition in a simulated environment. Successful implementation of PBT programmes promises to improve patient care and safety by allowing healthcare professionals to acquire proficiency in clinical skills without learning for the first time in clinical practice. To-date, the evidence around proficiency-based training in healthcare settings has concentrated on the acquisition of surgical skills.

**Aims:**

This scoping review aims to clearly determine the existing evidence relating to the use of PBT in the education of healthcare professionals. It will also identify existing evidence about PBT in the context of non-technical skills training.

**Methods:**

A sensitive search strategy will be employed across databases including PubMED, CINAHL, SCOPUS, the Cochrane Library, EMBASE and PsychINFO to look for material addressing the use of proficiency-based methodologies in the training of healthcare professionals. Trials registries will be searched for trials which are in progress but not yet reported. All evidence pertaining to proficiency-based simulation for healthcare professionals will be considered for inclusion. The search will be limited to information published in the English language. The titles and abstracts of identified works will be screened by at least two reviewers. Relevant works which are identified through screening will be retrieved in full text and reviewed for methodological consistency with PBT. Results will be presented in tabular form and as an evidence map and will be reported in accordance with PRISMA-ScR guidelines.

## Introduction

Simulation based training is being increasingly utilised for both technical and non-technical skill acquisition in healthcare with the aim of reducing medical error and patient harm [1–3]. The most common form of simulation-based training is recognised as an artificial, controlled, and safe environment that allows for the rehearsal of specific events. This form of simulation, referred to as immersive simulation or high-fidelity simulation (HFS), is not usually used as a form of summative assessment, but instead offers an enhanced formative learning experience through debriefing, expert feedback and participant reflection [4–7]. The endpoint of immersive simulation is to equip learners with knowledge and skills to enable them to tackle a situation independently, but it does not demand that participants demonstrate they can apply their knowledge and skills in practice.

One criticism of HFS is that there is a lack of evidence linking simulation training to changes in participant behaviour or improved patient outcomes [8]. Proficiency based training (PBT) aims to address this issue by linking success in training directly with exhibiting desired behaviours and determining those desired behaviours by observing the real-world performance of task experts.

PBT utilises a metric-based approach, whereby learners undertake simulated scenarios, as in HFS, but satisfactory completion of training is determined by the participant demonstrating their ability to perform at the standard of a benchmark derived from expert performances [9].

The derivation of an expert performance benchmark is a rigorous process. Generally, example performances of the task in question are recorded. The recordings are analysed, and a series of steps which must be performed in sequence to successfully complete the task are identified [10–12]. Specific errors which jeopardise the success of the task or put the patient at risk of harm are also identified at this stage. The steps and errors form what is referred to as a “task metric”. The metric is then examined via a Delphi process by subject matter experts. Content validity of the performance metric is established through real-world task analysis and expert consensus [13]. The performance benchmark is then derived by scoring expert performances using the metric. A novice-expert comparison study can be performed concurrently to provide evidence for the construct validity of the performance metric [13, 14]. If there is an observable, statistically significant difference between the performances of experts and novices, it can be inferred that the metric measures a candidate’s ability to perform the task.

In PBT, the task metric and the performance benchmark are shared with learners in advance of simulation to set expectations and goals. Sharing the metric and benchmark in advance also allows learners to engage in deliberate practice. Deviation from optimal performance can be identified and learners can practice those steps until they are able to meet the performance benchmark. Learners are then assessed in a simulated environment and are graded with the task metric against the benchmark that they have practiced with.

Proficiency-based progression (PBP) is a closely related concept in which progression through a training programme is linked to proficiency-based benchmarks and assessments. For the purposes of this review, PBP programmes are considered as a sub-set of PBT.

It is essential to consider the concepts of competency and proficiency to understand the differences between HFS and PBT. Medical education has traditionally aimed to train learners to the standard of competency. Competency in this context refers to the “minimally qualified test-taker” or “borderline test-taker”. This person has the minimum knowledge and skill deemed necessary to complete the task. Setting this minimum standard often involves use of robust statistical methods such as borderline regression, or variations on the Angoff method [15]. Despite this, there is often significant variability in passing standards between institutions [16–18].

Proficiency based training mandates that learners achieve beyond the level of competency, as the proficiency benchmark is set at the average performance level of task experts. Achieving the proficiency standard in simulation does not imply the candidate has the same performance capacity as an expert in real life settings. It demonstrates that candidates can achieve an expert standard in simulation before progressing to a clinical environment.

PBT has been demonstrated to improve skills acquisition and retention by both novice and experienced learners, with PBT learners significantly outperforming traditionally trained learners [19]. PBT methodology is well studied in the context of technical skills training, in surgical [14, 20, 21] and non-surgical specialties in medicine [22, 23], and is a distinct form of training from simulation assessed by other methods such as global rating scales and checklists without proficiency-based benchmarking.

Recently, the application of PBT in non-technical skill acquisition, such as communication, has demonstrated that can be a more effective method of teaching communication skills compared to traditional immersive simulation [24, 25]. This scoping review will identify the extant evidence relating to the use of PBT for both technical and non-technical skills training in healthcare.

In two recently published systematic reviews of PBT, search results were limited to clinical trials and randomised controlled trials. Both reviews used restrictive search strategies likely to exclude works which could not contribute to an accompanying meta-analysis [19, 26]. Additionally, a limited array of terms were used in each search strategy which may have excluded works that followed proficiency-based methodologies.

A search of Prospero and the Open Science Framework was conducted to identify ongoing reviews in the field. One systematic review in progress was identified [27]. The systematic review focused on the context of procedural skills training and aimed to evaluate whether published studies claiming to use proficiency-based progression employed all features of PBP.

In contrast with these recent and proposed systematic reviews, this scoping review [28, 29] differs in that it aims to identify all existing work pertaining to proficiency-based training in healthcare. This is useful as previous systematic reviews have not used inclusive search strategies and so the full extent of published work on proficiency-based healthcare is not clearly established.

### Review question

What is the extent of the evidence regarding proficiency-based training for healthcare professionals? Has this methodology been used to train non-technical skills? Where are there gaps in the evidence base where future research is required?

### Eligibility criteria

#### Participants

Any published studies examining PBT in the context of healthcare education will be considered, including but not limited to papers looking at the training of doctors, nurses, midwives, and other allied health professions.

#### Concept

All evidence pertaining to PBT in the training of healthcare professionals will be considered. Papers should demonstrate the design or use of proficiency-based methodology, by using a metric derived from expert performance to objectively assess candidate performance in simulations of the task of interest.

#### Context

All evidence pertaining to PBT in the training of healthcare professionals will be considered. This review will be limited to publications in the English language.

#### Types of sources

This scoping review will consider all evidence examining the use of PBT methodologies in the training of healthcare workers.

Studies that describe the design of PBT programmes will also be considered for inclusion.

In addition, systematic reviews that meet the inclusion criteria will also be considered and studies identified using citation pearl indexing.

Editorials and opinion papers will be considered for inclusion in this scoping review if they contribute to describing PBT methodology directly.

### Methods

#### Search strategy

A sensitive search strategy was constructed with the goal of identifying all extant evidence pertaining to proficiency-based training in healthcare. A pilot search of PubMed and CINAHL was made to identify relevant articles on the topic. We used keywords, index terms and common phrases to form the basis of a search strategy. The strategy will be tailored for use in 6 databases: PubMed, CINAHL, Cochrane Library, EMBASE, Scopus, and PsychINFO (see Appendix I). Each included work will be screened for references to further relevant sources of evidence.

Articles published in English will be included. There will be no limitation on date range for articles published to be included. Papers which are not written in English, or which claim to describe a proficiency-based training but do not conform strictly to proficiency-based methodology as outlined in our protocol will be excluded. Language limitations were chosen as our group lacks the resources required for extensive use of professional translation services.

We will search ClinTrials.gov and WHO ICTRP for registered trials of proficiency-based training that have not yet been reported.

#### Evidence selection

All identified works will be uploaded into *EndNote (Clarivate Analytics*,* PA*,* USA)*, and duplicates removed. Titles and abstracts will then be uploaded to Covidence (Covidence, VIC, Australia). Reviewers will be briefed and a pilot test conducted to ensure consistency between individuals during screening. Citations will be screened by at least two independent reviewers. Sources will proceed to full text screening if they purport to use a proficiency-based methodology or if the methods described in the abstract are consistent with proficiency-based training.

Retrieved full text works will be assessed against our inclusion and exclusion criteria by two reviewers. If a work is excluded after review of the full text, the reasons will be reported in the scoping review. Disagreements arising between reviewers will be resolved by discussion or via adjudication by an additional reviewer. A flow diagram as consistent with (PRISMA-ScR) [30] will outline the results of the search and screening processes.

#### Data extraction

Data from works that pass full text screening will be extracted by the reviewers using the data extraction tool presented in Appendix II. The data will include authorship, year of publication, type of study, aims of the study, details about study participants, trainee outcomes, and whether the study addresses the training of non-technical skills. There will be additional data points extracted from publications where they are relevant, including information about the structure and presentation of task metrics, methodology of metric development, definition of experts in the contexts of metric development and benchmarking, approach to benchmark derivation, types of simulation, components of validation addressed in the publication and patient-oriented outcomes where present.

The data extraction tool presented in Appendix II may be modified as necessary during the extraction process. Any modifications performed will be detailed and justified in final review. Where possible, the reviewers will attempt to contact authors to gather missing data.

### Data analysis and presentation

Evidence identified for inclusion will be presented in a tabular form, including authors, year of publication, and type of study, and aims of the study. An evidence map will be produced presenting in graphical form the type and number of evidence sources aligned to different purposes e.g. metric design, simulation integration, or trials against alternative training methods. This map will be divided to present evidence aimed at technical and non-technical skills. A narrative summary will accompany the tabulated and graphical results and will describe how the results relate to the review objective and questions. We expect two interesting outcomes from our evidence map will be an examination of the degree of interlinking between research groups publishing in the area, and an assessment of how many papers purport to examine proficiency-based training but fail to follow the requisite processes.

## Appendices

### Appendix I: Search strategy

Search Strategy and Logic grid – example search for pubmed.

Research Question.

What is the extent of the literature examining proficiency-based training progression for healthcare professionals? Has this methodology been used to train non-technical skills? Where are there gaps in the evidence base where future research is required?

Population: Healthcare Professionals.

Concept: Simulation training and clinical competence.

Context: Proficiency-based training programmes. Evidence published in the English language.

Pubmed.

**Table:**

POPULATION
“Healthcare profession*” OR Medic* OR Doctor* OR Physic* OR “general practitioners” OR Paediatric* OR Gynaecolog* OR Obstetri* OR Nurs* OR Midwif* OR Physiotherap* OR “Phsical therap*” OR “Occupational therap*” OR pharmac* OR “Radiation therap*” OR Radiograph* OR Dent* OR “Health Personnel“[Mesh]
CONCEPT
“Proficiency based simulation*” OR “Proficiency-based simulation*” OR “Proficiency based progression*” OR “Proficiency-based progression*” OR “Proficiency based training” OR “Proficiency-based training” OR “proficiency AND based AND simulation” OR “Proficiency AND based AND progression” OR Proficiency OR Proficient OR “Metric-based simulation” OR “Metric-based assessment” OR “Metric-based training” OR “Metric based simulation” OR “Metric based assessment” OR “Metric based training”
CONTEXT
“Simulation training” OR “Professional Competence” OR “Clinical competence” OR “Surgical simulation” OR “Simulation Training” OR “Simulation Training“[Mesh] OR “Professional Competence“[Mesh] OR “Clinical Competence“[Mesh]

### Appendix II: Data extraction instruments

Draft Data Extraction Tool – Stage 1.

1. Authors:
  - 1 st Author:
  - Author list in alphabetical order:
2. Year:
3. Type of publication:
4. Aims of publication:

Metric development/Validation/Benchmarking/Educational Measurement.

6) Metric Development Approach.
  - Recording/Task Analysis/Delphi Group/Expert opinion/Not applicable.
  - Stress testing/pilot study.
  - Delphi group composition: Experts [] Psychologist [] PPI [].
  - Delphi group composition: Multidisciplinary [] Single Specialty [].
  - Delphi group methodology – question development, endpoints definition, statistical methods.
7) Definition of experts.
8) Simulation modality.
  - Immersive sim/low-fidelity/virtual reality/trainer device/other.
9) Benchmark Derivation.
  - Concurrent with task analysis/separate study.
  - Statistical methods.
10) Trainee Outcomes.
11) Patient-oriented outcomes.
12) Skills focus: Technical Non-technical.
13) Metric validation (Kane’s Framework of Medical Validation).

Scoring [] Generalisation [] Extrapolation [] Implication/Decision [].

14) If measuring educational attainment:

Observational/Experimental/Quasi-experimental.
